# miR-124/VAMP3 is a novel therapeutic target for mitigation of surgical trauma-induced microglial activation

**DOI:** 10.1038/s41392-019-0061-x

**Published:** 2019-08-16

**Authors:** Yan Chen, Jing-xian Sun, Wan-kun Chen, Gen-cheng Wu, Yan-qing Wang, Ke-ying Zhu, Jun Wang

**Affiliations:** 1 Department of Integrative Medicine and Neurobiology, State Key Laboratory of Medical Neurobiology, School of Basic Medical Science, Fudan University, 200032 Shanghai, China; 2 Institutes of Brain Science, Collaborative Innovation Center for Brain Science, Fudan University, 200032 Shanghai, China; 3 Institute of Acupuncture and Moxibustion, Fudan Institutes of Integrative Medicine, Fudan University, 200032 Shanghai, China; 4 Department of Anesthesiology, Fudan University Shanghai Cancer Center, 200032 Shanghai, China; 5 Department of Oncology, Shanghai Medical College, Fudan University, 200032 Shanghai, China; 6 Department of Clinical Neuroscience, Center for Molecular Medicine, Karolinska Institutet, 17176 Stockholm, Sweden

**Keywords:** Neuroimmunology, Immunological disorders, Experimental models of disease

## Abstract

Activation of microglia and the subsequently elevated inflammatory cytokine release in the brain during surgery predispose individuals to cognitive dysfunction, also known as postoperative cognitive dysfunction (POCD). miR-124 is one of the most abundant microRNAs in the brain that regulates microglial function. Elucidating the role of miR-124 in microglial activation in the context of surgery may therefore promote understanding of as well as therapeutic development for post-surgical disorders involving microglial activation. The downstream targets of miR-124 were investigated using bioinformatic screening and dual-luciferase reporter assay validation, and vesicle-associated membrane protein 3 (VAMP3) was identified as a potential target. The kinetics of miR-124/VAMP3 expression was first examined in vitro in microglial cells (primary microglia and BV2 microglial cells) following lipopolysaccharide (LPS) stimulation. LPS induced a time-dependent decrease of miR-124 and upregulated the expression of VAMP3. Manipulating miR-124/VAMP3 expression by using miR-124 mimics or VAMP3-specific siRNA in LPS-stimulated BV2 microglial cells inhibited BV2 microglial activation-associated inflammatory cytokine release. To further examine the role of miR-124/VAMP3 in a surgical setting, we employed a rat surgical trauma model. Significant microglial activation and altered miR-124/VAMP3 expression were observed following surgical trauma. We also altered miR-124/VAMP3 expression in the rat surgical trauma model by administration of exogenous miR-124 and by using electroacupuncture, which is a clinically applicable treatment that modulates microglial function and minimizes postoperative disorders. We determined that electroacupuncture treatment specifically increases the expression of miR-124 in the hypothalamus and hippocampus. Increased miR-124 expression with a concomitant decrease in VAMP3 expression resulted in decreased inflammatory cytokine release related to microglial activation post-surgery. Our study indicates that miR-124/VAMP3 is involved in surgery-induced microglial activation and that targeting miR-124/VAMP3 could be a potential therapeutic strategy for postoperative disorders involving microglial activation.

## Background

Surgery is an important physical intervention to achieve certain medical needs. However, postoperative complications as a consequence of surgical stress, which is a systematic response including hormonal and metabolic changes following surgical trauma, are troublesome and could influence the quality of life for patients.^1,2^ Of note, postoperative cognitive dysfunction (POCD) is a significant complication after surgery that impairs learning, memory, and other cognitive functions.^3,4^ POCD occurs days to weeks after surgery and could become a persistent disorder, being associated with an increased mortality within the first 3 months post-surgery.^5^ Hence, strategies to better prevent and control the development of POCD are urgently needed.

Neuroinflammation, including microglial activation and concomitant inflammatory responses, is believed to contribute to the development of POCD.^6–9^ Microglia are tissue resident macrophages in the central nervous system (CNS) that constantly monitor the brain microenvironment, responding rapidly to even minor changes and becoming primed.^10^ The activation of microglia is often associated with the secretion of proinflammatory cytokines, including interleukin-1 (IL-1), IL-6, and tumor necrosis factor-α (TNF-α).^11^ These elevated levels of inflammatory cytokines will predispose individuals to mental or cognitive disorders.^12–14^ Previous studies have shown that surgery will induce the activation of microglia in the brain and increase the expression of inflammatory cytokines.^15,16^ However, how to control and inhibit the activation of microglia during surgical stress remains to be elucidated.

As one of the most abundant miRNAs in the brain, miR-124 is not only necessary for the development of the nervous system but is also considered a key regulator of the function of microglia/macrophages.^17,18^ The overexpression of miR-124 skews activated macrophages back to a resting state by inhibiting the expression of the myeloid cell transcription factor C/EBPα and PU.1.^19^ In addition, miR-124 limits cytokine production from lipopolysaccharide (LPS)-stimulated macrophages by targeting STAT3.^20^ However, the intracellular mechanism of miR-124 and its link to cytokine production have not been fully elucidated.

In the present study, we utilized the Gene Expression Omnibus (GEO) database and bioinformatic analysis to validate vesicle-associated membrane protein 3 (VAMP3) as one target of miR-124. We hypothesized that miR-124/VAMP3 participates in microglial activation following surgical trauma and that it is a therapeutic target to control surgical trauma-induced microglial activation. To test our hypothesis, we first performed in vitro experiments to identify the expression of miR-124/VAMP3 upon microglial activation and examined the release of inflammatory cytokines following miR-124 transfection or siRNA treatment. In vivo, we employed a rat surgical trauma model previously established in our lab to mimic surgical stress with significant microglial activation.^21^ We treated operated rats with either intracerebroventricular (i.c.v) injection of miR-124 or electroacupuncture (EA), which is a safe, clinically applicable treatment that was previously reported to regulate microglial function and to reduce surgical stress as well as postoperative complications.^22–26^ To our knowledge, this is the first study highlighting the importance of miR-124/VAMP3 in regulating postoperative microglial activation.

## Methods

### Screening for miR-124 targets

Gene expression profiles of three cell lines with ectopic overexpression of miR-124 in vitro were chosen: HEK 293T (GSE18837), HCT 116 (GSE25224), and mouse neuronal cells (GSE8498). The gene expression profiles GSE18837 (platform: GPL9494, SMD Print_1539 Homo sapiens), GSE8498 (platform: GPL1261, Affymetrix Mouse Genome 430 2.0 Array) and GSE25224 (platform: GPL10379, Rosetta/Merck Human RSTA Custom Affymetrix 2.0 microarray) were obtained from the NCBI (National Center for Biotechnology Information) GEO database (http://www.ncbi.nlm.nih.gov/geo/). The differentially expressed genes were analyzed using the GEO2R program. The top 250 differentially expressed genes were selected according to their *P* values.

### Dual-luciferase reporter assay

A 3′-UTR luciferase reporter vector of VAMP3 was made by cloning the 3′-UTR of the corresponding mRNA into the NdeI and EcoRI-HF sites of the pGL3-promoter vector. The primers for the 3′-UTR of VAMP3 mRNA were 5′-CGC CAT ATG TGA AGA ACC GAT GAA ACT GAA GCC C-3′ (forward) and 5′-CGG AAT TCT ACC CAG CAT CTT AAA TAC AGC GG-3′ (reverse). All vectors were confirmed by sequencing analysis. HEK 293T cells were cotransfected with luciferase reporter plasmid and the miR-124 mimics or negative control (NC). After 24 h, luciferase activities were measured using a Dual-Luciferase Reporter Assay System (Promega, USA) according to the manufacturer’s instructions.

### Isolation of microglial cells from the CNS

Mononuclear cells were isolated from the CNS of Sprague-Dawley (SD) rats as previously described.^27^ In brief, 12 h after surgical stress, rats were anesthetized and perfused transcardially with saline. The hypothalami of three rats were pooled and homogenized, and mononuclear cells were subsequently isolated using 40/70% discontinuous Percoll gradients. We harvested ~8.5 × 10^4^–1.25 × 10^5^ microglia from 3 hypothalami.

### Primary microglial culture

Primary cultures of microglial cells were generated from mixed cell cultures of the forebrains of 0- to 1-day-old SD rats as described previously.^28^ Mixed glial cultures contained oligodendrocytes, microglia, and astrocytes. After 10 days in culture, the mixed glial cultures were shaken for 2 h at 220 rpm. The supernatants containing 90% microglia were plated onto uncoated tissue culture plates. Fifteen minutes after plating, non-adherent cells (predominantly astrocytes and oligodendrocytes) were removed by three washes with PBS. Microglia were maintained in DMEM/F12 medium with 10% FBS. Microglial cells were seeded at a density of 1 × 10^6^ cells in 1 ml or 0.5 ml in a 12-well or 24-well tissue culture plate. The purity of the microglial culture was almost 99%, as determined by immunocytochemical staining of Iba1 (1:400 dilution; 019–19741, Wako Chem Co., Japan; data not included).

### BV2 cell transfection

The BV2 murine microglial cell line was obtained from the Cell Bank of the Chinese Academy of Science (Shanghai, China) and cultured in Dulbecco’s modified Eagle’s medium (DMEM) supplemented with 10% FBS, 100 IU/ml penicillin, and 100 μg/ml streptomycin sulfate. Cells were maintained in a humidified atmosphere of 5% CO_2_ at 37 °C and transfected with 50 nM miR-124, inhibitor or control mimics (Ruibo Biotechnology, China) using Lipofectamine 2000 (Invitrogen, America) according to the manufacturer’s instructions. Twenty-four hours after transfection, the cells were treated with 100 ng/ml LPS for the indicated times.

### Animals

Experiments were performed on adult male Sprague–Dawley (SD) rats (Experimental Animal Center, Shanghai Medical College of Fudan University, China) weighing 200–220 g. Rats were housed in a temperature-controlled (22 ± 2 °C) and light-controlled (12:12 h light–dark cycle) room with free access to food and water. Prior to experimental manipulation, rats were allowed to acclimatize to the housing facilities for 1 week. All experimental protocols and animal handling procedures were approved by the Animal Care and Use Committee (ACUC) of Fudan University, which are consistent with the National Institutes of Health Guide for the Care and Use of Laboratory Animals (DHEW Publications, NIH, 80–23).

### Surgical trauma model

The surgical trauma model was performed as previously described.^21,27^ In brief, rats were anesthetized with pentobarbital sodium (40 mg/kg; i.p). Animals were then incised longitudinally to a length of 6 cm along the dorsal median line and 5 cm along the abdominal median line, with intestinal tracts taken out from the abdominal cavity and exposed for 5 min. After surgery, the wounds were sutured, and the animals were kept warm under standard housing conditions.

### Electroacupuncture administration

According to others and our previous study, bilateral ‘Zusanli (ST36)’ acupoints were selected for EA treatment.^21,29,30^ In brief, two pairs of stainless steel needles of 0.3 mm diameter were inserted perpendicularly at a depth of 5 mm into the bilateral acupuncture points ST 36, located 5 mm below and lateral to the anterior tubercle of the tibia. Each pair of needles was connected with the output terminals of an EA apparatus (Model LH202H, Huawei Co., Beijing, China). To prevent the tissue from adapting to the same frequency of EA, 2.5 s sparse (2 Hz) and dense (15 Hz) pulse alternation was used, and the alternation interval was 5 s. The intensity was adjusted to induce slight muscle contraction of the hind limb (≤2 mA). EA stimulation was applied immediately after the surgery and lasted for 30 min. EA treatment started immediately after surgery while the rats were still anesthetized. Therefore, rats in the control group only received surgery, but they were also placed on the same working bench for the same period of time when the others group received electroacupuncture treatment.

### Tissue preparation and immunofluorescence

Rats were anesthetized with pentobarbital sodium (40 mg/kg; i.p) and transcardially perfused 24 h after surgical stress with PBS (0.1 M phosphate buffer, pH 7.4, containing 0.9% NaCl) followed by 4% paraformaldehyde. Brains were removed and fixed in the same fixative solution for another 4 h and cryoprotected in 20% sucrose. Brain tissues were sectioned (25 μm) using a cryotome (Leica 2000, Germany). The sections were stored in antifreeze solution (30% sucrose and 30% ethylene glycol in PBS) in a −20 °C freezer until further use. Standard fluorescent immunohistochemistry was performed using primary antibody against Iba1 (1:400; Wako Bio-products) and fluorescent secondary antibodies (FITC-conjugated, green, 1:1000, Invitrogen, USA). Immunofluorescent images were captured using an Olympus FV1000 fluorescence microscope and analyzed with ImageJ software.

### RNA extraction and quantitative RT-PCR

Total RNA containing miRNAs and mRNAs was extracted from isolated microglia, cell lines, or primary microglial culture using TRIzol reagent (Invitrogen, America) following the manufacturer’s instructions. The purity and concentration of the RNA were tested by NanoDrop before reverse transcription. The relative abundance of target mRNAs was quantified with SYBR Green qRT-PCR detection (iCycler iQ® real-time PCR detection system, Bio-Rad, CA, USA). The sequences of primers for each target mRNA are as follows: VAMP3, Forward: 5′-ACC TCA CAA CTT TGG TGC TG-3′, Reverse: 5′-CAT TCC CAG CTA AAT GCA CA-3′; ITGB1, Forward: 5′-GCA AAT GCC AAA TCT TGC GGA-3′, Reverse: 5′-CCA TCC CTT TGC TGC GAT TG-3′; SERP1, Forward: 5′-GCG AGT CCG AGA GGT TCT T-3′, Reverse: 5′-CGC GCT GAG TTA TGT TCT TG-3′; GAPDH, Forward: 5′-CCC TTC ATT GAC CTA AAC TAC-3′, Reverse: 5′-CTT CTC CAT GGT GGT GAA GAC-3′. To analyze miRNA expression, specific stem-loop reverse transcription primers and forward/reverse primers were designed by BioTNT (Shanghai, China). Endogenous control (U6) was used as an internal reference for standardization. Relative quantification was performed by determining the n-fold differential expression using the 2^−ΔΔCt^ method and expressed as % of U6. Melting curves were used to estimate the purity of the amplified band. All experiments were performed in triplicate.

### Immunoblotting

Following treatment, cultured cells were washed twice with ice-cold PBS and lysed in radioimmunoprecipitation assay lysis buffer (RIPA buffer, Beyotime, Shanghai, China). For in vivo experiments, the brain was quickly removed 24 h post-surgery and placed on an ice-cold surface. The hypothalamus and hippocampus were dissected, snap-frozen in liquid nitrogen and stored at −80 °C until processing. Tissues were ultrasonically homogenized in RIPA at 12,000 rpm centrifugation for 10 min, and the supernatant was collected for western blot analysis and enzyme-linked immunosorbent (ELISA) assay. Equal amounts of protein (50 μg each sample) were analyzed using SDS-PAGE. Following transfer to polyvinylidene difluoride (PVDF) membranes, the membranes were blocked for 2 h in 5% nonfat milk at room temperature and were subsequently incubated with primary antibodies against VAMP3 (1:1000, Cell Signaling Technology, USA) or glyceraldehyde-3-phosphate dehydrogenase (GAPDH, 1:10000, Earthox, USA) overnight at 4 °C. Membranes were then incubated with horseradish peroxidase-conjugated secondary antibody (1:10,000, Earthox, America) for 1 h at room temperature. Protein bands were visualized using a chemiluminescent detection system (Tian Neng, China) and captured using a Quant LAS 4000 mini. Immunoreactive bands were quantitatively analyzed using ImageJ software.

### Enzyme-linked immunosorbent assay (ELISA)

Cell supernatants or brain tissues were assayed for TNF-α and IL-6 proteins using ELISA. Twenty-four hours after stimulation, the culture supernatants were harvested and stored at −70 °C. For in vivo experiments, the samples were prepared as indicated in the immunoblotting section. The concentrations of IL-6 and TNF-α were measured using enzyme-linked immunosorbent assay (ELISA) kits as described in the manufacturer’s instructions (R&D Systems, USA).

### Intracerebroventricular injection of chemicals

Implantation of the cannula was performed under sodium pentobarbital anesthesia (40 mg/kg; i.p). Rats were fixed in a stereotaxic apparatus under anesthesia, and a stainless steel guide cannula (0.5 mm in diameter) with an inserted cannula (0.25 mm in diameter) was implanted into the right lateral ventricle (−0.5 mm AP, −1.5 mm ML, −4.5 mm DV to the bregma; according to *the Rat Brain in Stereotaxic Coordinates* by George Paxinos and Charles Watson) and fixed onto the skull with dental cement. Implantation was conducted at least 1 week prior to the surgical stress. The miRNA-124 (2 μg in 50 μl PBS, PM10691; Applied Biosystems, Carlsbad, CA, USA) or control miRNA (2 μg in 50 μl PBS, AM17110; Applied Biosystems) was mixed with transfection reagent (Lipofectamine 2000; Invitrogen, Paisley, UK), diluted to the appropriate concentration (3 μl in 50 μl PBS) and applied during 30 s via the cannula in a volume of 10 μl 6 h before surgery. At the end of each experiment, the position of the cannula was verified.

### Image analysis

Western blot bands and immunofluorescence staining (integrated optical density, IOD) were analyzed using ImageJ software. The quantitative statistical graphs were created based on the relative fold change.

### Statistics

Data are presented as the mean ± standard error of the mean (SEM). Statistical analysis was performed using an unpaired *t*-test or one-way ANOVA followed by the LSD post-test or Bonferroni post-test using GraphPad Prism software. A *P* < 0.05 value was considered to be statistically significant.

## Results

### VAMP3 is a direct mRNA target of miR-124

**Fig. 1:**
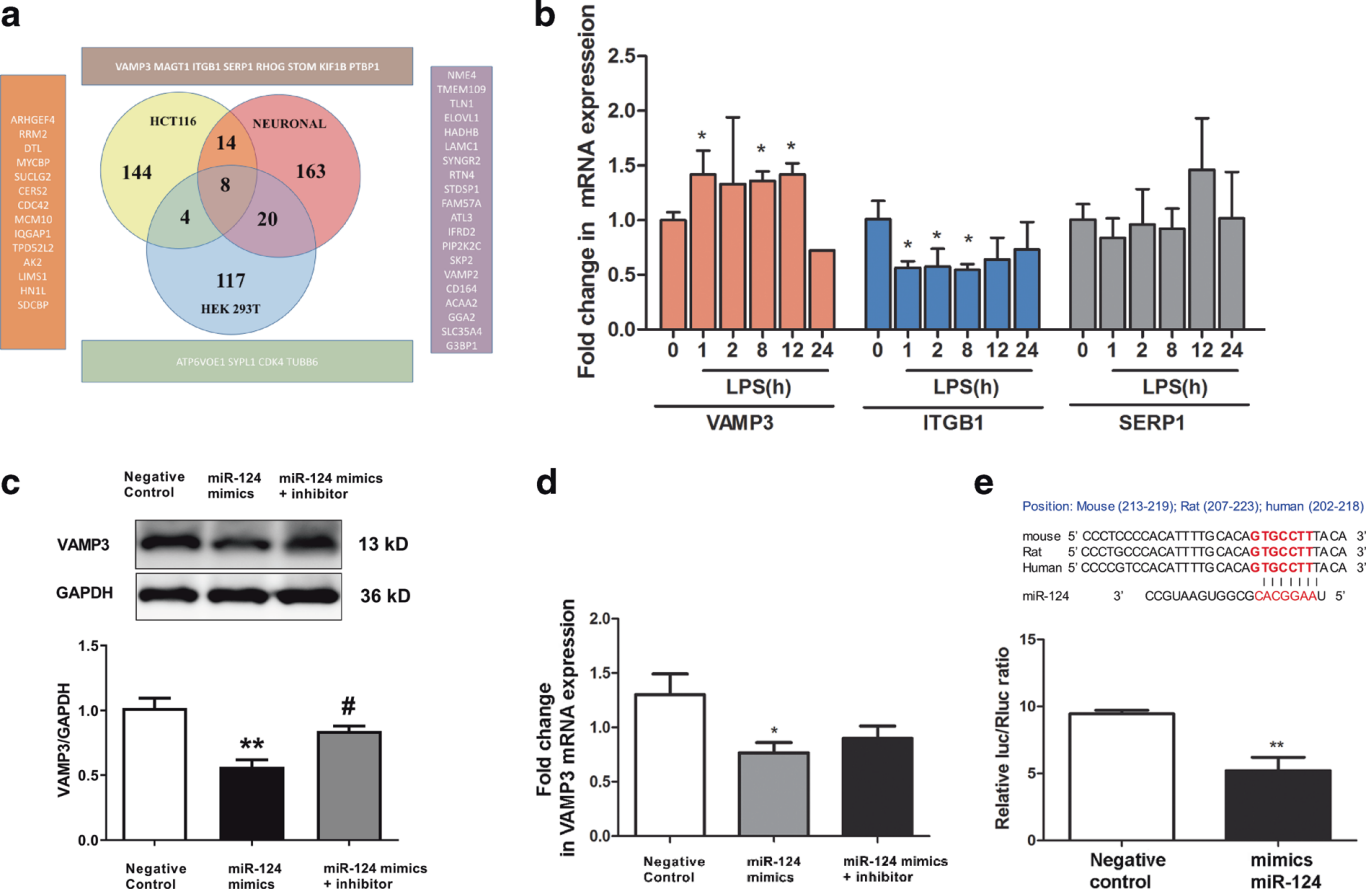
VAMP3 is a direct target of miR-124. **a** Overlapping genes from three cell lines overexpressing miR-124. The datasets from three cell lines (HEK 293T, HCT 116, and neuroblastoma CAD) overexpressing ectopic miR-124 were analyzed with the GEO2R tool, and the top 250 differentially expressed genes were included. **b** mRNA expression of VAMP3, ITGB1, and SERP1 was detected by RT-PCR assays after LPS stimulation in BV2 microglial cells for the indicated hours; *P < 0.05, compared to unstimulated cells, *n* = 4. **c** VAMP3 protein expression 24 h after transfection with miR-124 mimics, miR-124 mimics + inhibitor, or negative control; *P < 0.05, compared to the negative control, *n* = 3. **d** mRNA expression of VAMP3 24 h after transfection with miR-124 mimics, miR-124 mimics + miR-124 inhibitor or negative control; *P < 0.05, compared to the negative control, *n* = 3. **e** Alignment of potential binding sites for miR-124 in the 3′UTRs of VAMP3 mRNA. Renilla/firefly luciferase activity was measured 24 h after cotransfection of plasmids with miR-124 mimics or control mimics; **P < 0.01, *n* = 3

To identify the downstream targets of miR-124, we made use of published expression profiles from different cell types with miR-124 overexpression. The cell lines with ectopic overexpression of miR-124 in vitro were chosen, which are HEK 293T (GSE18837), HCT 116 (GSE25224) and mouse neuronal cells (GSE8498). Each dataset was analyzed using GEO2R software, and the top 250 differentially expressed genes were selected according to their *P* value (supplementary data). The overlap of the three different cell types revealed 8 genes (*VAMP3, ITGB1, SERP1, RHOG, PTBP1, MAGT1, KIF1B*, and *STOM*) that were downregulated in miR-124-overexpressing cells (Fig. 1a). Intriguingly, *VAMP3, ITGB1*, and *SERP1* have previously been associated with the inflammatory response,^31–33^ so we further examined the mRNA expression of these three genes in activated BV2 microglial cells (Fig. 1b). Real-time PCR data revealed that mRNA expression of VAMP3 (the vesicle-associated membrane protein 3) was stably increased after LPS stimulation; the expression of ITGB1, which is an identified downstream target of miR-124 and associated with microglia proliferation and function,^34–36^ was significantly decreased after microglial activation. However, whether VAMP3 is directly regulated by miR-124 remains to be validated, and there is no study linking VAMP3 and microglia activation to date. In vitro gain-of-function experiments were performed by transfecting BV2 microglial cells with miR-124 mimics. The protein level and mRNA expression of VAMP3 decreased significantly after transfection with miR-124 mimics (Fig. 1c, d). The expression of miR-124 and VAMP3 in response to LPS treatment revealed an inverse correlation between them, which indicates that VAMP3 might be a potential target of miR-124. Furthermore, a dual-luciferase reporter assay was utilized to verify the targeted binding sites of VAMP3. The potential binding sites of miR-124 in the 3′-UTR of the VAMP3 mRNA were determined using TargetScan. We cloned the VAMP3 3′-UTR sequence into a reporter vector pGL3. Luciferase activity of the pLuc-VAMP3 3′-UTR unit was significantly inhibited after transfection with the miR-124 mimic (Fig. 1e). These data confirmed that miR-124 negatively regulates VAMP3 expression directly via binding to its mRNA 3′-UTR.

### The expression of miR-124/VAMP3 is altered during microglial activation

**Fig. 2:**
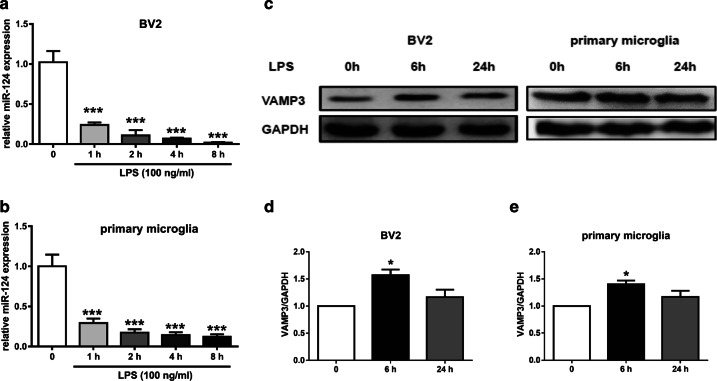
Kinetics of miR-124/VAMP3 expression after LPS-induced microglial activation (primary microglia and BV2 cell line). **a**, **b** Expression of miR-124 was detected by RT-PCR in both the murine BV2 microglial cell line (**a**) and primary microglia (**b**) after LPS stimulation for the indicated hours; ***P < 0.001, compared to the non-stimulated control group, *n* = 3. **c** Protein expression of VAMP3 after LPS stimulation for the indicated hours, and the statistics of VAMP3 expression in BV2 cells and primary microglia are shown in **d** and **e**, respectively; *P < 0.05, compared to the non-stimulated counterpart control, *n* = 3. These data are representative of two independent experiments with similar results

As one of the most abundant miRNAs in the CNS, miR-124 is essential for microglial quiescence. We therefore wondered how miR-124 is affected by microglial activation. We examined the kinetics of miR-124 expression in both primary microglia and BV2 microglial cells from 1–8 h following LPS stimulation and observed a time-dependent decrease in miR-124 expression in both cells (Fig. 2a, b). Consistent with the increased mRNA expression of VAMP3 after LPS stimulation (Fig. 1b), an upregulated protein expression of VAMP3 in primary microglia and BV2 microglial cells was also observed after LPS stimulation for 6 h, and it returned to baseline after 24 h (Fig. 2c–e). These data indicate that miR-124/VAMP3 dynamically responds to microglial activation.

### Targeting miR-124/VAMP3 inhibits cytokine release subsequent to LPS stimulation in vitro

**Fig. 3:**
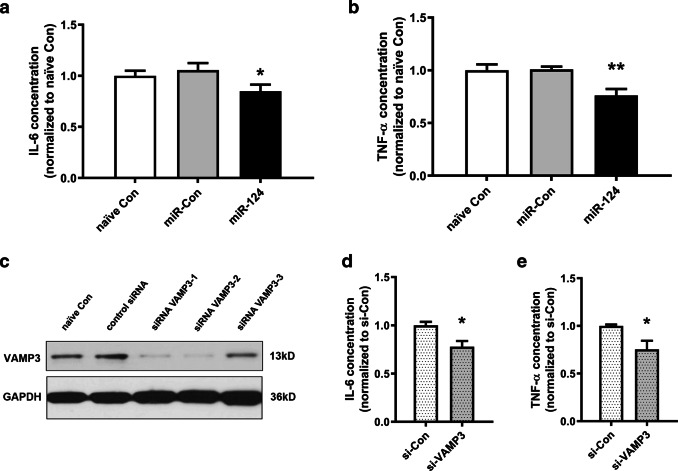
Targeting miR-124/VAMP3 inhibits cytokine release subsequent to LPS stimulation in vitro. **a**, **b** BV2 microglial cells were transfected with miR-124 mimics or control mimics or untransfected (naïve control) for 6 h, followed by 100 ng/ml LPS stimulation overnight. Supernatants were collected for ELISAs for IL-6 (**a**) and TNF-α (**b**); *P < 0.05, **P < 0.01, compared to naïve control and control mimics, *n* = 3. **c** The VAMP3 silencing effect of the siRNAs was detected by performing western blotting to detect VAMP3 protein expression in BV2 microglial cells. siRNA-VAMP3-2 showed the best silencing effect and was used to silence VAMP3 for the following experiments. **d**, **e** The release of the proinflammatory cytokines IL-6 and TNF-α was detected by ELISAs after VAMP3 silencing; *P < 0.05, compared to the non-silenced control group (si-Con), *n* = 3. Data are from one experiment representing two independent experiments

Next, we wanted to investigate whether altered expression of miR-124/VAMP3 is the consequence of microglial activation or whether it could also determine the inflammatory outcome by artificially manipulating the expression of miR-124 or VAMP3. To understand the causality, we modulated miR-124 expression by transfection of the miR-124 mimic or control miRNA (miR-Con). We determined that overexpression of miR-124 in BV2 microglial cells strongly decreased the secretion of IL-6 and TNF-α compared with cells treated with the scrambled miRNA, as detected by ELISAs (Fig. 3a, b). To further clarify the function of VAMP3 in activated microglia, small interfering RNA (siRNA) was used to knock down VAMP3 protein expression. The efficiency of interference was validated, and among the siRNA candidates for VAMP3 knockdown, siRNA-VAMP3-2 (siVAMP3) was selected for subsequent experiments (Fig. 3c). We treated BV2 microglial cells with siVAMP3, and as expected, VAMP3 silencing significantly repressed the secretion of cytokines in activated BV2 microglial cells (Fig. 3d, e). Therefore, targeting miR-124/VAMP3 could modulate inflammatory responses after microglial activation.

### miR-124/VAMP3 mediates microglial activation in a rat surgical stress model

**Fig. 4:**
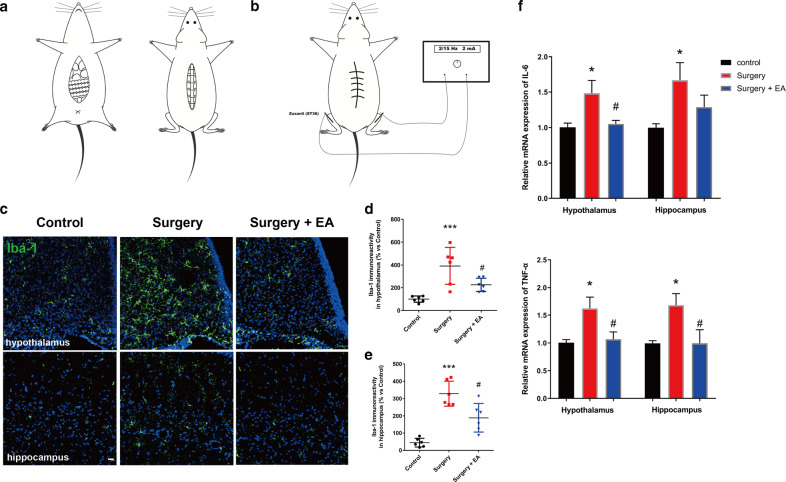
Electroacupuncture mitigates microglial activation in a rat surgical trauma model. **a** Simplified diagram of the rat surgical trauma model. Rats were cut open longitudinally to a length of 6 cm along the dorsal median line and 5 cm along the abdominal median line with exposure of intestinal tracts for 5 min. **b** Electroacupuncture was performed on the bilateral Zusanli acupoint (ST36) for 30 min right after surgery; the stimulating intensity was 2 mA with a frequency of 2/15 Hz. **c** Immunofluorescence staining of microglia using Iba1 antibody, graph bar = 20 μm. **d**, **e** Relative quantification of the optical density of Iba1 staining in the hypothalamus (**d**) and hippocampus (**e**). **f** mRNA expression of IL-6 and TNF-α in the hypothalamus and hippocampus was detected by RT-PCR; *P < 0.05, compared to control rats, ^#^*P* < 0.05, compared to surgical rats, *n* = 6 in each group. Data represent one experiment from three independent experiments

Electroacupuncture (EA), a modified acupuncture therapy, is a potent treatment to control systematic inflammation via multiple targets and is clinically available with few side effects.^30,37,38^ Although the direct mechanism of EA remains unknown, it is believed that EA activates the neural-immune reflex circuit and targets dopaminergic receptors to confer anti-inflammatory properties. EA was widely reported to relieve surgery-related symptoms and protect organ function during both the perioperative period and postoperation, including postoperative cognitive impairment.^39–42^ EA also strongly suppresses microglial activation in a variety of animal models.^23,43^ Herein, we employed EA treatment (2/15 Hz, 2 mA) immediately after surgery for 30 min (Fig. 4b) in our rat surgical stress model (Fig. 4a). This surgical procedure led to increased microglial activation in the brain, notably in the hypothalamus and hippocampus, which are the brain regions highly relevant to POCD. EA treatment significantly inhibited the activation of microglia (Fig. 4c, d).

**Fig. 5:**
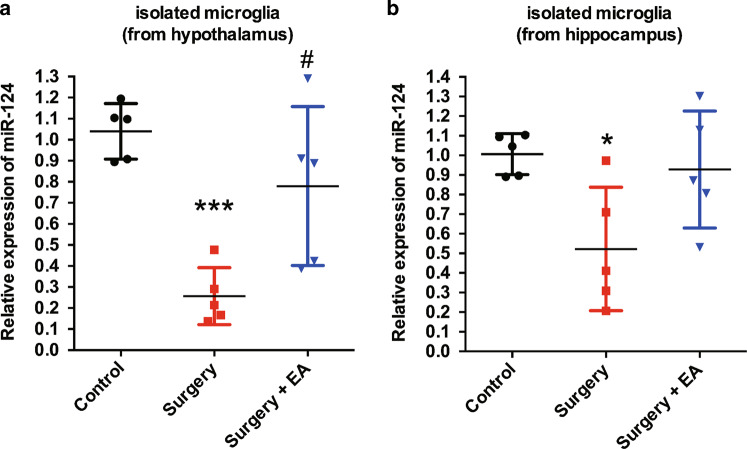
miR-124 expression in brain microglia after surgical trauma and EA treatment. miR-124 expression was detected by RT-PCR from isolated microglia from the hypothalamus (**a**) and hippocampus (**b**); *P < 0.05, ***P < 0.001, compared to the control group, ^#^*P* *<* 0.05, compared to the surgery group. Data represent one experiment from three independent experiments

**Fig. 6:**
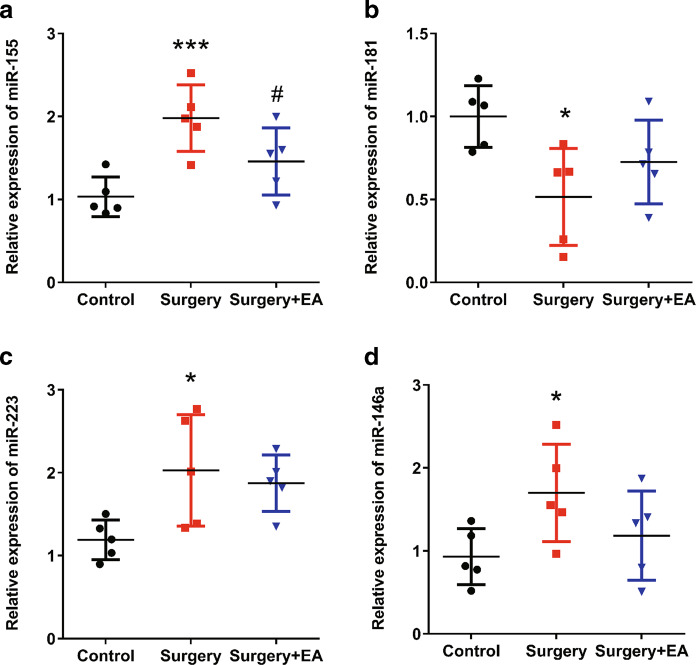
Expression of other neurimmiRs in the hypothalamus after surgical trauma and EA treatment. **a**–**d** The expression of miR-155, miR-181, miR-223, and miR-146a; *P < 0.05, ***P < 0.001, compared to the control group, ^#^*P* *<* 0.05, compared to the surgery group. Data represent one experiment from three independent experiments

We also examined the mRNA expression of genes related to microglial activation and inflammation in the hypothalamus and hippocampus. The expression of IL-6 and TNF-α was significantly increased post-surgery but was downregulated in the EA-treated group (Fig. 4f). Along with microglial activation following surgical stress, we also observed an obvious miR-124 reduction in microglia isolated from the hypothalamus and hippocampus (Fig. 5a, b), which was similar to the response of microglial cells undergoing LPS stimulation in vitro. This finding indicates a robust interaction between miR-124 and microglial activation both in vitro and in vivo. EA treatment also upregulated miR-124 expression (Fig. 5a). The expression of some other miRNAs related to neuroinflammation (also known as neurimmiRs) was also altered after surgical stress (Fig. 6a-d), including miR-155, miR-181, miR-146a, and miR-223, and EA treatment modulated their expression, especially miR-155 expression (Fig. 6a).

**Fig. 7:**
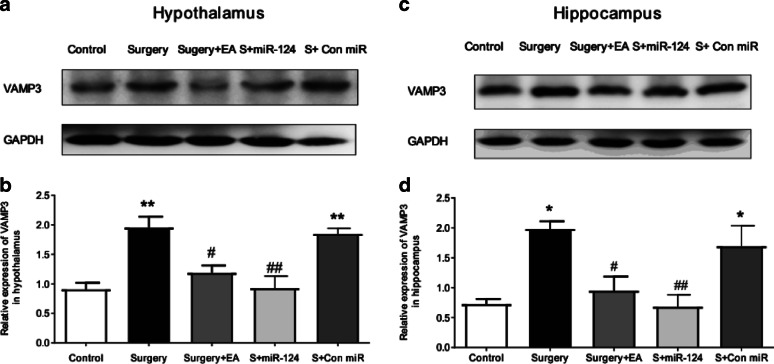
The protein expression of VAMP3 is increased after surgery and is modulated by EA and exogenous miR-124 administration. **a**, **b** VAMP3 protein expression in the hypothalamus was detected by western blots. **c**, **d** VAMP3 protein expression in the hippocampus. *P < 0.05, **P < 0.01, compared to the control group; ^#^*P* < 0.05, ^##^*P* < 0.01, compared to the surgery group; *n* = 6. Data are representative of one experiment from three independent experiments

**Fig. 8:**
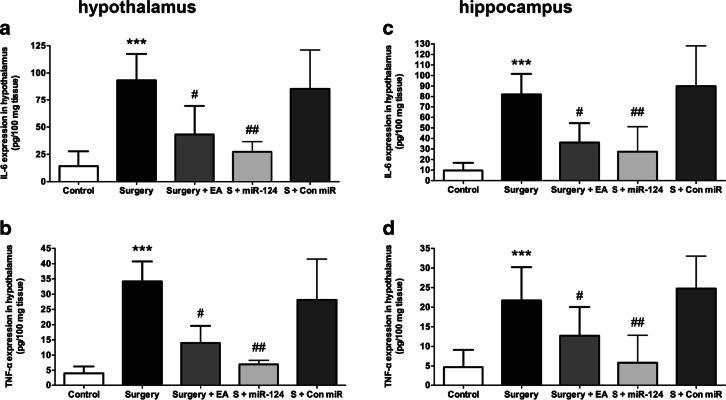
Inflammatory cytokine release is increased after surgery and is mitigated by EA or exogenous miR-124 administration. **a**, **c** The expression of IL-6 in the hypothalamus and hippocampus was detected by ELISAs. **b**, **d** The expression of TNF-α in the hypothalamus and hippocampus. ***P < 0.001, compared to the control group; ^#^*P* < 0.05, ^##^*P* < 0.01, compared to the surgery group; *n* = 5. Data represent one experiment from three independent experiments

Next, we examined the expression of the miR-124 downstream target VAMP3 by western blotting and evaluated inflammatory cytokine expression (IL-6 and TNF-α) by ELISAs. In addition to the EA treatment group, we used another therapeutic group as a positive control in which rats were pretreated with 100 ng miR-124 (i.c.v) before surgery. Both EA treatment and miR-124 treatment effectively downregulated the expression of VAMP3 in both the hypothalamus (Fig. 7a, b) and hippocampus (Fig. 7c, d), and this led to a strong inhibition of IL-6 and TNF-α (Fig. 8a, b). Therefore, miR-124/VAMP3 mediates the therapeutic effect of EA on surgery-induced microglial activation, and targeting miR-124/VAMP3 inhibits microglial activation-associated inflammation post-surgery.

## Discussion

The present study demonstrates an important role of miR-124 and its direct downstream target VAMP3 in the release of proinflammatory cytokines from activated microglia, both in vitro under LPS stimulation and in vivo during surgical stress. Furthermore, we provide evidence that electroacupuncture ameliorates surgical stress-induced microglial activation via enhancing miR-124 synthesis, consequently preventing VAMP3-mediated proinflammatory cytokine release in the hypothalamus and hippocampus. Targeting miR-124/VAMP3 either by EA or exogenous miR-124 administration can reduce microglial activation-mediated neuroinflammation.

Inflammatory cytokines produced by activated microglia may result in impaired synaptic plasticity and neuronal apoptosis.^44–46^ In the context of surgery, microglial activation is a predisposing factor underlying the development of post-surgery psychiatric disorders, especially POCD.^9,47^

The elucidation of molecular mechanisms that regulate the production of inflammatory cytokines in microglia is crucial to the understanding of neuroinflammation and to identify potential targets for therapeutic interventions for POCD. Several studies have addressed the important role of miRNAs in regulating microglial development and activation.^48,49^ A relatively high level of miR-124 maintains the quiescence of microglia. Conversely, a decrease in microglial miR-124 fails to retain microglia in a resting state and tends to skew microglia to a more proinflammatory phenotype.^19^ By analyzing GEO datasets and miRNA target prediction websites, we identified VAMP3 as one of the direct targets of miR-124. Overlapping gene analysis using the datasets the same species as the animal model we used in the study (rat) instead of using datasets from other species would be preferable. However, there are very few omics data from *Rattus*, and the surgical trauma model we have established is more potent and stable in rats. Preparation of a microarray directly from our microglial cells would also be useful. However, because there was already a public dataset based on many other cell types that we could make use of, we used the public dataset to obtain results rapidly and cost-effectively.

In macrophages and synoviocytes, VAMP3, a recycling endosomal SNARE, mediates the transport of cytokines to the cell surface.^31,50^ Loss or inactivation of any SNARE component (such as VAMP3) will dampen the secretion of cytokines (e.g., TNF-α and IL-6) that require the delivery of recycling endosomes to fuse with the cell membrane, and most of the accumulated presynthesized cytokines may then undergo degradation processes. Double immunostaining of VAMP3 and some markers of recycling endosomes and elucidation of the underlying mechanism through which VAMP3 regulates cytokine release would strongly enhance our results. However, since endosomes are tiny compartments in cells, we did not achieve any satisfactory results by coimmunostaining of VAMP3 with other markers. Nevertheless, previous studies have already validated the localization of VAMP3 in recycling endosomes and elaborated on how VAMP3 regulates cytokine release.^51^

Our results revealed that VAMP3 protein levels were upregulated in LPS-stimulated BV2 microglial cells, along with increased inflammatory cytokine release, and that knockdown of VAMP3 could significantly reduce the secreted IL-6 and TNF-α levels. Our findings support the notion that proinflammatory cytokine secretion from microglia requires VAMP3. Although BV2 microglial cells are not a true representative of primary microglia, they exhibit the basic functions of microglia, such as response to LPS stimulation. In addition, BV2 microglial cells are easier to use for miR-124 transfection and siRNA intervention experiments with than are primary microglia.

We also noticed that VAMP3 expression increased at 6 h but returned to normal levels at 24 h. We believe that the regulation of protein expression is a complicated biological process largely determined by certain important molecules, but numerous pathways and mediators might also be involved. In addition, VAMP3 is a key component of recycling endosomes and regulates the delivery of cytokines to the outside of the cell surface. However, because the mRNA production of cytokines such as TNF-α and IL-6 in microglia will return to baseline as early as 4–8 h after LPS priming,^52^ there could be a decreased demand for recycling endosomes. Therefore, accumulated VAMP3 would undergo protein degradation, and less VAMP3 would be detected at 24 h.

Microglia are highly sensitive to environmental perturbations.^53^ Acute or chronic stress results in structural remodeling of microglia, and it enhances the release of proinflammatory cytokines in the brain.^54^ While alterations of microglial functions by chronic stress are well documented, the impact of acute stressors on microglial activity has long been ignored. Sugama et al. found that microglia were rapidly activated by cold stress, characterized by a robust increase in CD11b immunoreactivity in the brain.^55^ Blandino and Frank also confirmed that acute stress significantly modulates microglial activity.^56,57^ In the current study, we observed an enhanced density of Iba-1 immunoreactivity, together with increased release of inflammatory cytokines in the brain (hypothalamus and hippocampus) 24 h post-surgery, which further supports the idea that acute stressors (surgical trauma) can modulate microglial structure and function. We also have unpublished data regarding the cognitive impairment of surgical rats. These results did not show an acquired quick response to dangerous stimuli in the shuttle box avoidance test at days 1 and 7 post-surgery, whereas normal rats quickly learned to avoid the danger (data not included in the manuscript).

The rat surgical trauma model was previously established in our lab, and we have reported that EA treatment is an effective therapeutic intervention to modulate microglial function.^21,23,58^ As evident in the current study, the surgical trauma model is characterized by significant microglial activation, and EA treatment strongly inhibited it. We screened potential miRNAs involved in microglial activation and focused on miR-124 because it is highly relevant to the function of microglia. Intrathecal administration of miR-124 reversed persistent hyperalgesia by modulating microglial activation.^59^ A recent study also observed that intracranial administration of miR-124 reduces the proinflammatory phenotype of activated microglia and promotes behavioral improvement in ischemic mice.^60^ We noted that the expression of other neurimmiRs (miR-155, miR-181, miR-223, and miR-146a) was also altered in our surgical trauma model. These neurimmiRs could be potential therapeutic targets for POCD and could be used as a readout to evaluate or predict the efficacy of a certain treatment to control surgery-induced microglial activation. miR-124 is also highly expressed in neurons. To avoid the contamination of neuronal-derived miR-124, its expression was quantified in isolated microglia from the hypothalamus and hippocampus rather than bulk brain tissue in our study.

Electroacupuncture (EA) is a modified acupuncture therapy combined with electric stimulation and has been utilized more frequently than regular acupuncture treatment in recent years for neurological conditions, including stroke, depression, insomnia, and stress-related disorder.^61–63^ We have previously reported that EA suppressed proinflammatory cytokine release both in the spleen and plasma.^21,27^ Herein, we further demonstrated that EA can modulate microglial activation and inhibit subsequent proinflammatory cytokine production in the hypothalamus following surgical trauma. While emerging studies using experimental animal models suggest that EA may be effective in regulating inflammatory responses, the underlying mechanism and pathway remain poorly elucidated. In our study, we used EA treatment as a therapeutic intervention, based on its reported function and good clinical application, to assess the importance of miR-124/VAMP3 in regulating microglial activation after surgical stress. We also provide a potential mechanism underlying the therapeutic effect of EA. Our results reinforce the theoretical basis or rationale of EA in postoperative management, but in regard to treating patients, a proper treatment protocol must be determined for each individual, and clinical trials are needed to assess the long-term effects.

In summary, administration of exogenous miR-124 or increasing miR-124 by a therapeutic intervention, along with downregulated VAMP3 expression, controls microglial activation and subsequent inflammatory responses. miR-124/VAMP3 is a novel therapeutic target for surgery-related microglial activation intervention, and this provides information for related drug development or other therapies. Treatments with the ability to modulate miR-124/VAMP3 in microglia could be applied to manage postoperative disorders involving microglial activation.

## Conclusions

Taken together, our data demonstrate that VAMP3 is a direct downstream target of miR-124. miR-124/VAMP3 underlies microglial activation after surgery, and it is a potential therapeutic target for post-surgical disorders related to microglial activation.

## Data Availability

The data used and/or analyzed during the current study are available from the corresponding author on reasonable request.
